# Biological Role of *Nardonella* Endosymbiont in Its Weevil Host

**DOI:** 10.1371/journal.pone.0013101

**Published:** 2010-10-01

**Authors:** Takashi Kuriwada, Takahiro Hosokawa, Norikuni Kumano, Keiko Shiromoto, Dai Haraguchi, Takema Fukatsu

**Affiliations:** 1 Okinawa Prefectural Plant Protection Center, Naha, Japan; 2 National Institute of Advanced Industrial Science and Technology (AIST), Tsukuba, Japan; University of Poitiers, France

## Abstract

Weevils constitute the most species-rich animal group with over 60,000 described species, many of which possess specialized symbiotic organs and harbor bacterial endosymbionts. Among the diverse microbial associates of weevils, *Nardonella* spp. represent the most ancient and widespread endosymbiont lineage, having co-speciated with the host weevils for over 125 million years. Thus far, however, no empirical work on the role of *Nardonella* for weevil biology has been reported. Here we investigated the biological role of the *Nardonella* endosymbiont for the West Indian sweet potato weevil, *Euscepes postfasciatus*. This insect is an experimentally tractable pest insect that can easily be reared on a natural diet of sweet potato root as well as on an agar-based artificial diet. By larval feeding on an antibiotic-containing artificial diet, *Nardonella* infection was effectively eliminated from the treated insects. The antibiotic-treated insects exhibited significantly lighter body weight and lower growth rate than the control insects. Then, the antibiotic-treated insects and the control insects were respectively allowed to mate and oviposit on fresh sweet potatoes without the antibiotic. The offspring of the antibiotic-treated insects, which were all *Nardonella*-negative, exhibited significantly lighter body weight, smaller body size, lower growth rate and paler body color in comparison with the offspring of the control insects, which were all *Nardonella*-positive. In conclusion, the *Nardonella* endosymbiont is involved in normal growth and development of the host weevil. The biological role of the endosymbiont probably underlies the long-lasting host-symbiont co-speciation in the evolutionary course of weevils.

## Introduction

Bacterial endosymbionts are found among diverse insects. Some obligate endosymbionts are obviously mutualistic and contribute to the fitness of their hosts, whereas other facultative endosymbionts are parasitic and often affect their hosts negatively [1]–[3]. In particular, the most intimate mutualistic associations are found in obligate endocellular bacteria like *Buchnera* in aphids and *Wigglesworthia* in tsetse flies. In these insects, the microbes are harbored in specialized cells called bacteriocytes or mycetocytes. In the insect bodies, these cells often constitute a conspicuous symbiotic organ called bacteriome or mycetome. The microbes are vertically transmitted to the next host generation in the maternal body at an early stage of oogenesis or embryogenesis, where the endosymbiont transmission is integrated into the developmental process of the host insects [4], [5]. In many obligate endosymbiont lineages such as *Buchnera* in aphids [6], *Carsonella* in psyllids [7], *Portiera* in whiteflies [8], *Baumannia* in sharpshooters [9], *Sulcia* in diverse homopterans [10], *Blochmannia* in carpenter ants [11], *Wigglesworthia* in tsetse flies [12], *Riesia* in primate lice [13], *Nardonella* in weevils [14] and others, the endosymbiont phylogeny generally mirrors the host phylogeny, suggesting host-endosymbiont co-speciation over evolutionary time.

These circumstances strongly suggest that these obligate endosymbionts play essential roles for their host insects. Thus far, however, experimental evidence for endosymbiont roles has been limited, mainly because of the fastidious nature of the microbes [15], [16]. In the *Buchnera*-aphid and *Blochmannia*-ant associations, it was demonstrated that the endosymbionts supply essential amino acids that are deficient in plant sap or sap-derived diets of the host insects [17], [18]. In the *Wigglesworthia*-tsetse and *Riesia*-louse associations, it was shown that the endosymbionts provide B vitamins that are scarce in vertebrate blood for the host insects [19], [20]. There are a number of reports that antibiotic treatments resulted in deleterious effects on survival, growth and reproduction of various insects, presumably by suppressing their obligate endosymbionts [21]–[23]. On the other hand, recent genomic approaches have opened a new window to look into potential biological roles of these endosymbionts. Even when physiological data are unavailable, gene contents of drastically reduced endosymbiont genomes often provide an insightful picture as to how an endosymbiont can metabolically contribute to its host insect. For *Buchnera*, *Blochmannia*, *Wigglesworthia* and *Riesia*, the genomic data corroborated the experimental results of nutritional provisioning [24]–[27]. In sharpshooters, it was suggested that *Sulcia* provides essential amino acids while *Baumannia* supplies cofactors [28], [29]. In cicadas, it was proposed that *Sulcia* synthesizes essential amino acids whereas *Hodgkinia* is specialized for cobalamin production [30]. Meanwhile, there are several cases wherein such genomic inferences do not clearly make sense. In the *Carsonella*-psyllid association, it was difficult to infer plausible biological roles of the endosymbiont whose genome is as small as 160 kilobases [31]. In the *Blattabacterium*-cockroach association, although the endosymbiont had been suggested to be involved in uric acid recycling [32], its genome encoded no genes for uricolytic enzymes [33]. Hence, for understanding of the biological roles of insect endosymbionts, not only genomic but also experimental approaches are needed, and for that purpose, an experimentally tractable model insect species carrying the endosymbiont of interest is of crucial importance.

**Figure 1:**
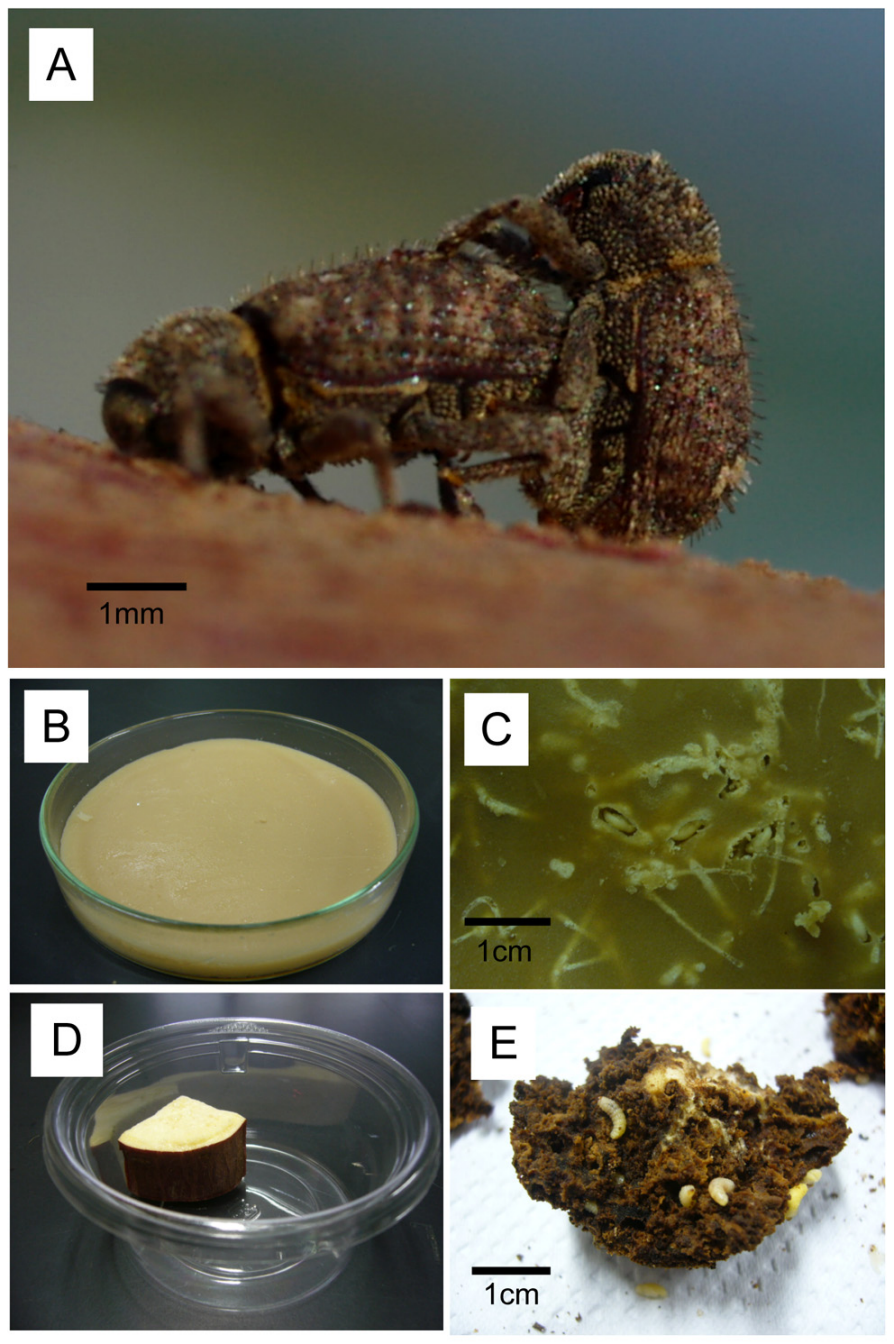
Rearing systems for *E. postfasciatus*. (A) A mating pair of *E. postfasciatus*. (B) An artificial diet rearing dish. (C) Larvae of *E. postfasciatus* in the artificial diet. (D) A sweet potato rearing container. (E) Larvae of *E. postfasciatus* in a piece of sweet potato.

Weevils constitute the most species-rich animal group with over 60,000 described species [34], [35], many of which possess specialized symbiotic organs and harbor bacterial endosymbionts therein [4], [36]. Among the diverse microbial associates of weevils, *Nardonella* spp. represent the most ancient and widespread endosymbiont lineage, having co-speciated with the host weevils for over 125 million years [14], [37], [38]. However, the endosymbiont roles for the host weevils are unknown: neither experimental works nor genomic data have been available to date. The West Indian sweet potato weevil, *Euscepes postfasciatus* (Fig. 1A), is a notorious pest of sweet potatoes worldwide [39]. In Okinawa, Japan, rearing techniques incorporating artificial diet have been developed to support an eradication effort for *E. postfasciatus*, based on sterile insect release [40]–[43]. Recently, we identified *Nardonella* infection and ovarial transmission in *E. postfasciatus* [38], which prompted us to investigate biological roles of the endosymbiont by making use of the experimentally tractable pest weevil species.

## Materials and Methods

### Insect

The laboratory colony of *E. postfasciatus* used in this study was originally collected in Yaese, Okinawa, Japan in November 2004, and has been maintained on sweet potato roots under the conditions of 25±1°C, 50–90% relative humidity and a long day regime (14 h light and 10 h dark) at the Okinawa Prefectural Plant Protection Center in Naha, Okinawa, Japan. An endosymbiont of the genus *Nardonella* was identified from the insect colony while no other endosymbiotic bacteria such as *Wolbachia*, *Rickettsia*, *Spiroplasma* and *Sodalis* were detected [38].

### Rearing on artificial diet

For antibiotic treatments, we used an agar-based artificial diet for *E. postfasciatus* consisting of sweet potato powder, sugar, soybean protein, cellulose, yeast, agar, vitamins and other ingredients [44]. Antibiotics were added to milled artificial diet (ca. 45°C) and kneaded with an aliquot of water. In the main experiments, 0.003% rifampicin treatment was adopted, while 0.3% and 0.03% rifampicin treatments were also tested in preliminary experiments. Normal artificial diet was used as control treatment. About 25 g of the diet was poured into each Petri dish (9.0 cm in diameter, 2.0 cm in depth) and allowed to solidify (Fig. 1B and C). Five scratch lines were made on the surface of the diet, and 50 eggs of *E. postfasciatus* per dish were inoculated on the lines using a tiny brush that was sterilized with 70% ethanol. The detailed procedures of egg collection and treatments were as described [42], [43]. The Petri dishes were maintained under the conditions of 25±1°C, 70 to 80% relative humidity and a long day regime (14 h light and 10 h dark).

### Rearing on sweet potato

Newly emerged adult weevils were collected from each of the artificial diets for the antibiotic treatment and the control treatment. Pairs of a male and a female were randomly picked from each of the artificial diets. Each of the pairs was introduced into a 200 ml plastic container with a piece of sweet potato (ca. 15 g), and allowed to mate and oviposit for seven days (Fig. 1D and E). After removal of the adult insects, the sweet potato containers were kept under the conditions of 25±1°C, 70–80% relative humidity and a long day regime (14 h light and 10 h dark) until adult emergence.

### Fitness measurements

The number of adult weevils that emerged from each of the artificial diet dish and the sweet potato containers was counted. The developmental time of the insects, in terms of days needed for adult emergence, was recorded for the first 5 adult weevils that emerged from each of the artificial diet dish and the sweet potato containers. The wet body weight of the insects was quantified after 10–14 days after adult emergence using an electronic balance (Libror AEX-120; Shimadzu, Kyoto, Japan). The left elytra length of the insects was measured under a binocular microscope (Leica MZ6).

### Diagnostic PCR

DNA samples were prepared from dissected abdomens of the adult insects using the QIAamp DNA Mini Kit (Qiagen) and subjected to diagnostic PCR detection of *Nardonella* endosymbiont. A 0.8 kb region of the 16S rRNA gene was amplified with the primers 16SA1 (5′-AGA GTT TGA TCM TGG CTC AG-3′) and 16S729R (5′-CGT GAA TAA GTG TCA GTC TTC A-3′) under the temperature profile of 95°C for 10 min followed by 35 cycles of 94°C for 30 sec, 55°C for 1 min, and 72°C for 1 min. To check the quality of the DNA preparations, a 0.6 kb segment of mitochondrial *cytochrome oxidase I* gene was amplified with the primers LCO1490 (5′-GGT CAA CAA ATC ATA AAG ATA TTG G-3′) and HCO2198 (5′-TAA ACT TCA GGG TGA CCA AAA AAT CA-3′) under the temperature profile of 94°C for 4 min followed by 35 cycles of 94°C for 30 sec, 48°C for 1 min, and 72°C for 1 min. The PCR products were electrophoresed on 2% agarose gels, stained with ethidium bromide, and observed on a UV transilluminator.

### Statistics

We used generalized linear mixed models [45] with R 2.9.2 software [46] for statistical analyses. The Petri dishes in the artificial diet experiments and the containers in the sweet potato experiments were treated as replicates, respectively. We used a Wald test to evaluate the statistical significance of each coefficient in the model.

## Results and Discussion

### Optimization of antibiotic treatment

In an attempt to establish the experimental procedure to eliminate *Nardonella* infection from *E. postfasciatus*, we performed a preliminary rearing of the insects on artificial diets containing different doses of rifampicin, an antibiotic that has been used for removal of endosymbiont infections in a diverse array of insects [21], [23], [47]. While few insects grew to adulthood on the diets containing 0.3% and 0.03% rifampicin, many adult insects emerged from the diet containing 0.003% rifampicin. Diagnostic PCR assay indicated that *Nardonella* infection was cured in these adult insects. The emergence rate (20%) was statistically not different from the emergence rate on the control diet (34%) (Table 1). In the following experiments, therefore, we adopted the 0.003% rifampicin diet for antibiotic treatment of *E. postfasciatus*.

**Table 1:** Effects of rifampicin doses in the artificial diet on adult emergence of *E. postfasciatus*.

Treatment	Antibiotic dose	No. of adults	Emergence rate
Rifampicin	0.3%	0	0.0%*
Rifampicin	0.03%	1	2.0%*
Rifampicin	0.003%	10**	20.0%
Control	0%	17	34.0%

* Significantly different from the control (*P<*0.05; Fisher's exact probability test).

** All were *Nardonella*-negative.

### Effects of antibiotic treatment on artificial diet

**Figure 2:**
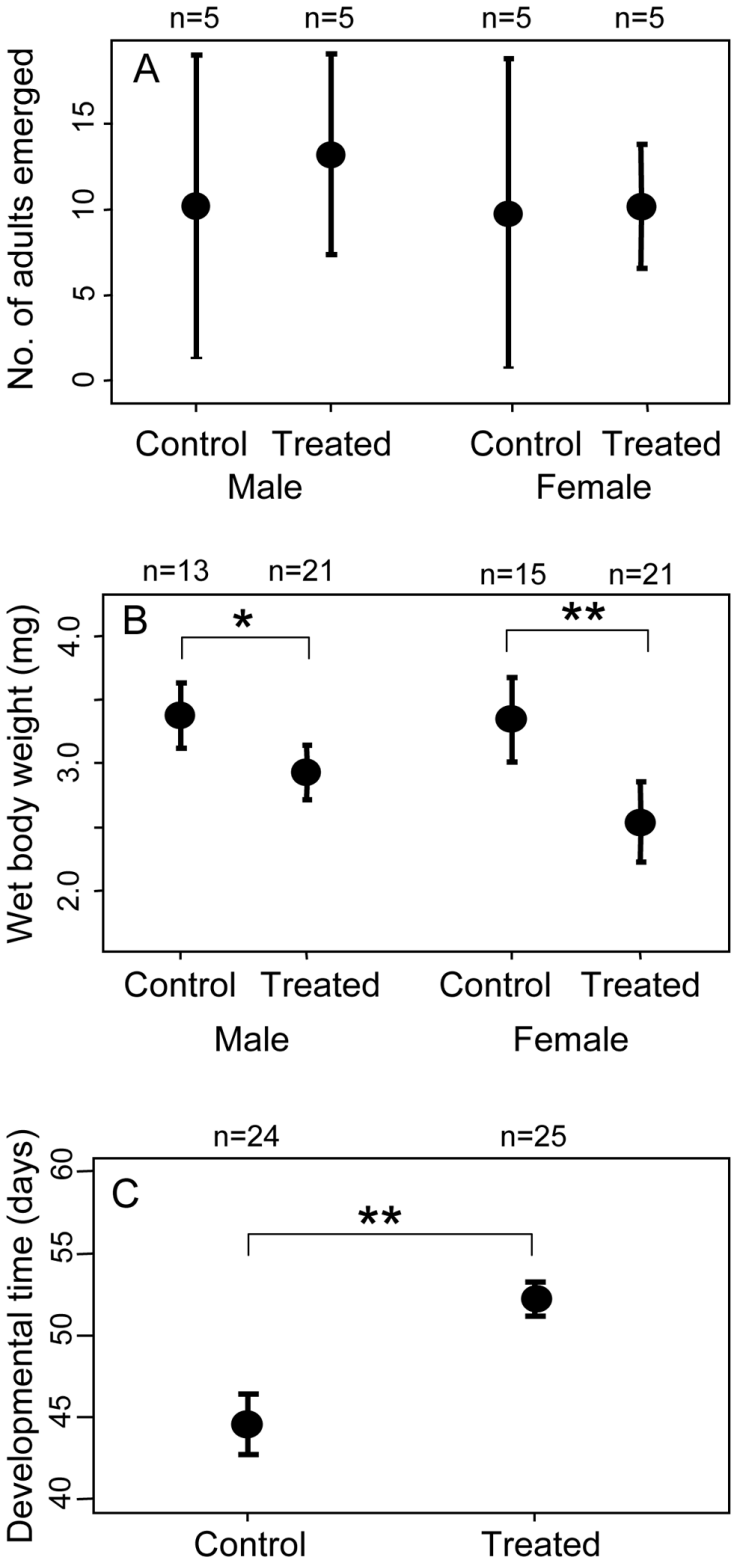
Effects of antibiotic treatment on fitness components of *E. postfasciatus*. (A) Adult emergence, (B) body weight and (C) developmental time. The insects were reared on artificial diets with and without 0.003% rifampicin. Means, standard deviations and sample sizes are shown. Asterisks indicate statistically significant differences (* *P*<0.05; ** *P*<0.01).

We inoculated 50 eggs of *E. postfasciatus* onto each of five dishes of the 0.003% rifampicin diet and five dishes of the control diet, and collected and inspected adult insects that emerged from these dishes. Number of the insects that attained adulthood was not different between the antibiotic treatment and the control treatment (Fig. 2A). By contrast, adult body weight and growth rate of the insects were significantly lower in the antibiotic treatment than in the control treatment (Fig. 2B and C). Diagnostic PCR assay revealed that most of the rifampicin-treated insects were *Nardonella*-negative while most of the control insects were *Nardonella*-positive (Table 2). These results suggested that removal of *Nardonella* infection negatively affected the growth and development of *E. postfasciatus*.

**Table 2:** *Nardonella* infection in adult insects of *E. postfasciatus* after rifampicin treatment.

	0.003% rifampicin treatment*	Control treatment*
1^st^ generation**	3.3% (1/30)	94.9% (37/39)***
2^nd^ generation**	0.0% (0/10)	100% (17/17)

* Percentage of *Nardonella* infection (number of *Nardonella*-positive insects/number of insects examined).

** In the 1^st^ generation, *E. postfasciatus* was reared on artificial diet with/without the antibiotic. In the 2^nd^ generation, *E. postfasciatus* was reared on sweet potato without the antibiotic.

*** Two *Nardonella*-negative insects were males. Male-specific degeneration of endosymbiotic system has been known from diverse insects such as weevils, lice, mealybugs and others [4], [54], [55].

### Effects of endosymbiont removal on natural diet

**Figure 3 pone-0013101-g003:**
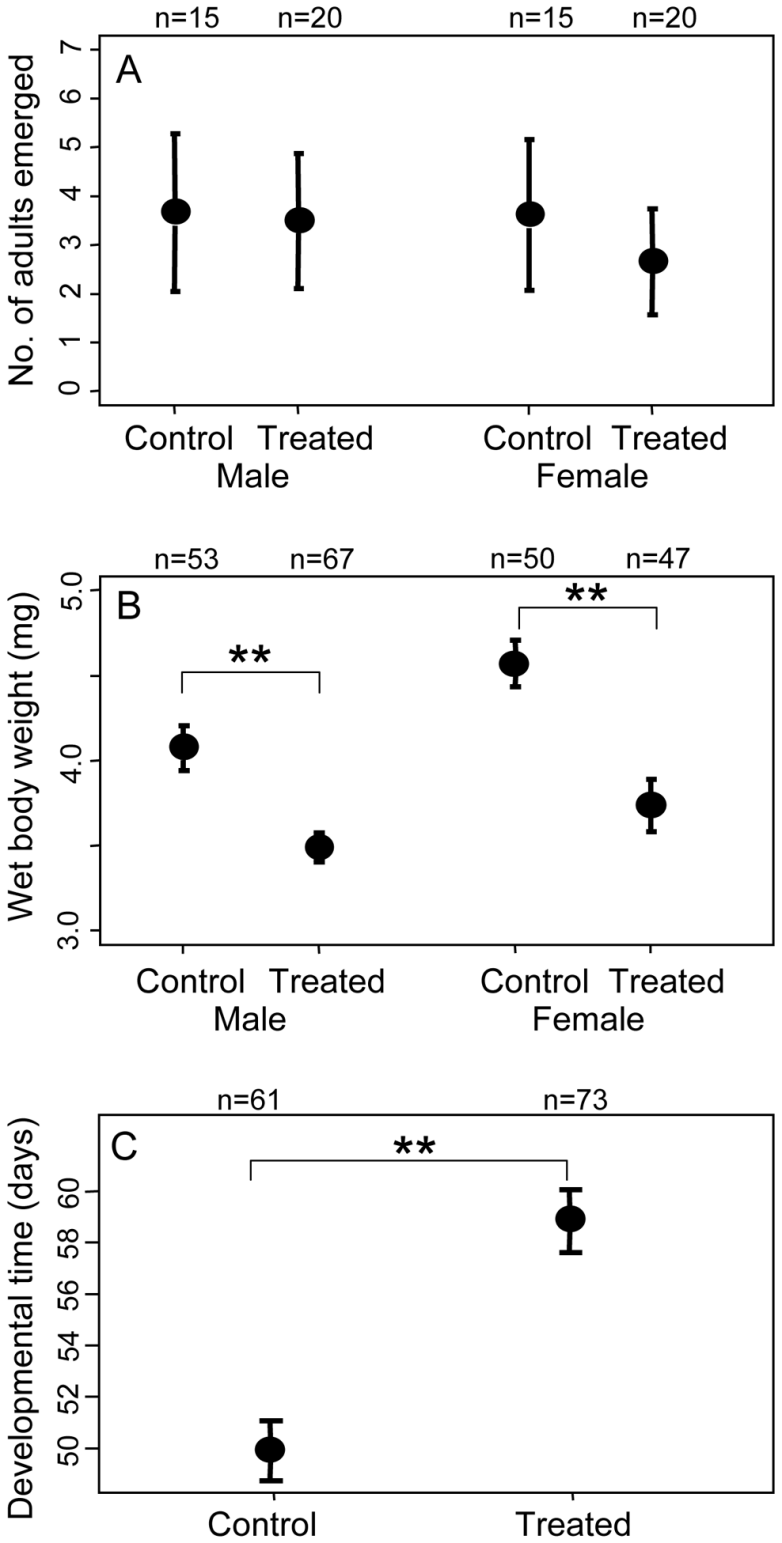
Effects of *Nardonella* elimination on fitness components of *E. postfasciatus*. (A) Adult emergence, (B) body weight and (C) developmental time. The insects were reared on sweet potatoes without the antibiotic. Means, standard deviations and sample sizes are shown. Asterisks indicate statistically significant differences (** *P*<0.01).

**Figure 4:**
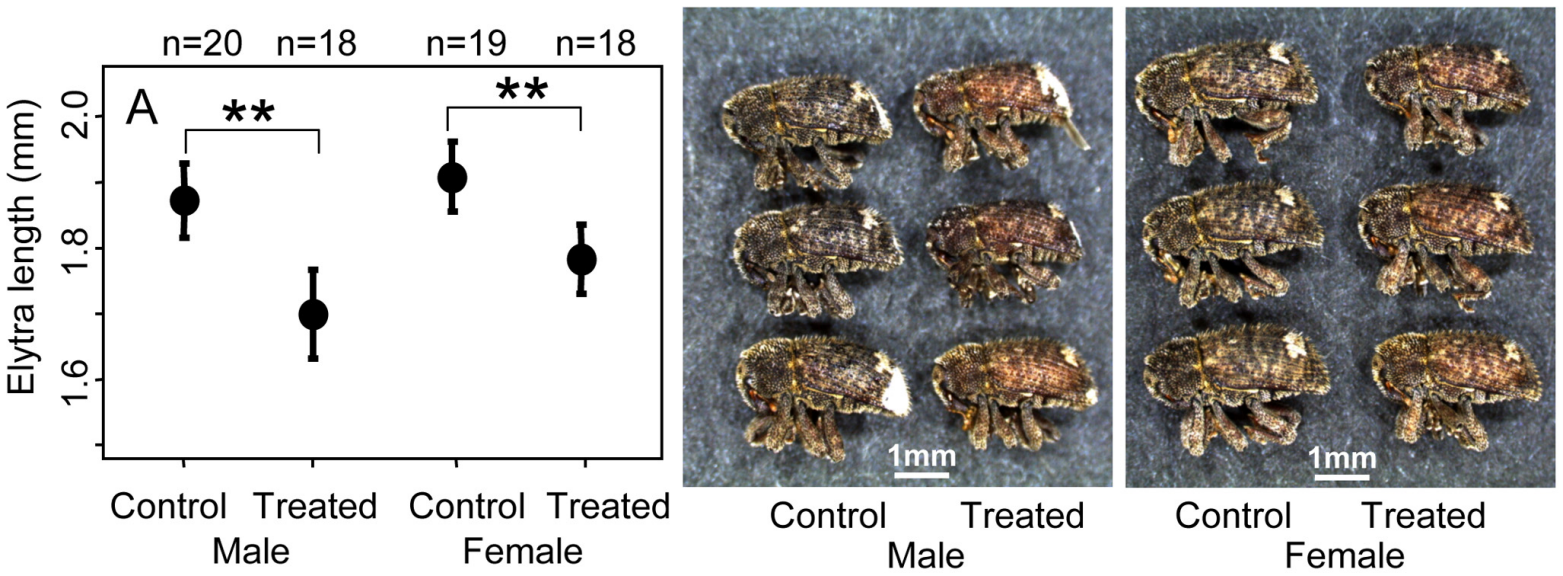
Effects of *Nardonella* elimination on adult body size and coloration. (A) Elytra length, (B) appearance of adult males and (C) appearance of adult females. The insects were reared on sweet potatoes without the antibiotic. In (A), means, standard deviations and sample sizes are shown, and asterisks indicate statistically significant differences (** *P*<0.01).

In the above experiments, we could not rule out the possibility that the antibiotic itself rather than the endosymbiont removal negatively affected the insects. In order to sort out the confounding factors, the following experiments were performed. The *Nardonella*-free insects that had emerged from the 0.003% rifampicin diet and the *Nardonella*-infected insects that had emerged from the control diet were respectively allowed to mate and oviposit on a piece of fresh sweet potato, and reared on the natural diet in the absence of the antibiotic. Number of the insects that attained adulthood was not different between the offspring of *Nardonella*-free insects and the offspring of *Nardonella*-infected insects (Fig. 3A). Meanwhile, adult body weight and growth rate of the insects were significantly suppressed in the offspring of *Nardonella*-free insects in comparison with the offspring of *Nardonella*-infected insects (Fig. 3B and C). The offspring of *Nardonella*-infected insects were larger in size and darker in color, whereas the offspring of *Nardonella*-free insects were smaller in size and reddish in color (Fig. 4). Diagnostic PCR assay confirmed that all the offspring of *Nardonella*-free insects were *Nardonella*-negative whereas all the offspring of *Nardonella*-infected insects were *Nardonella*-positive (Table 2).

### Involvement of *Nardonella* in host growth and development

All these results taken together, we concluded that the *Nardonella* endosymbiont is involved in normal growth and development of the host weevil *E. postfasciatus*. This study provides the first experimental evidence of *Nardonella*-weevil mutualism, while molecular phylogenetic studies have suggested obligate and mutualistic nature of the association on account of the long-lasting host-symbiont co-cladogenesis [14], [37]. Similar symptoms were observed in *Sitophilus* grain weevils deprived of their *Sodalis*-allied endosymbionts, including retarded larval growth, soft adult body and light cuticle color [48].

### Physiological role of *Nardonella* for host weevil

At present, the physiological role that *Nardonella* plays for the host weevil is unknown. The main food of *E. postfasciatus,* sweet potato root, is extremely carbohydrate-rich in the form of starch while relatively poor in proteins, lipids and some vitamins [49], [50]. It is conceivable that *Nardonella* synthesizes and provides the host with essential amino acids and/or vitamins to compensate for the nutritional deficiency, as *Buchnera* and *Wigglesworthia* do for their host aphids and tsetse flies, respectively [17], [19], [24], [25]. In the rice weevil *Sitophilus oryzae*, it was suggested that the *Sodalis*-allied endosymbiont provides B vitamins for the host insect [48], [51]. Considering that the genus *Sitophilus* is nested within the clade of diverse *Nardonella*-associated dryophthorid weevils [14], [37], it appears plausible that the *Nardonella* endosymbiont plays similar physiological roles for the host weevils, and the biological roles have been taken over by the *Sodalis*-allied endosymbiont in the lineage of the genus *Sitophilus*. To investigate such nutritional aspects in detail, a nutritionally-defined artificial diet should be developed for *E. postfasciatus*. Genome sequencing of *Nardonella* will provide a complementary and straightforward approach to gain insights into the biological roles of the endosymbiont for the host weevil.

### Sex-related effects

Table 3 summarizes the sex-related effects of *Nardonella* infection on *E. postfasciatus*. Sex ratio was affected neither by antibiotic treatment in the 1^st^ generation nor by presence/absence of *Nardonella* infection in the 2^nd^ generation. Number of emerged adults was influenced neither by *Nardonella* infection, by sex nor by their interaction, in both the 1^st^ and 2^nd^ generations. Elytra length in the 2^nd^ generation was significantly affected by *Nardonella* infection as shown in figure 4A, whereas neither sex nor interaction had significant effects on the trait. Wet body weight was significantly affected by *Nardonella* infection as shown in figures 2B and 3B. Sex exhibited a significant effect on wet body weight only in the 2^nd^ generation, probably because of larger sample size in the 2^nd^ generation than in the 1^st^ generation (see Fig. 2B and 3B), and reflecting generally larger body size in females than in males (see Fig. 4). Interaction between *Nardonella* infection and sex had no significant effect on wet body weight. In conclusion, no remarkable sex-related effects of *Nardonella* infection were detected in this study.

**Table 3:** Effects of *Nardonella* infection, sex, and interaction between them on fitness components of *E. postfasciatus* analyzed by generalized linear mixed models.

Treated generation	Fitness component	*Nardonella* infection***	Sex	Interaction
1^st^ generation*	Sex ratio	*z* = 0.77, *P* = 0.44	-	-
	No. of adults	*z* = 0.38, *P* = 0.71	*z* = 0.51, *P* = 0.61	*z* = 0.77, *P* = 0.44
	Wet body weight	*t*_64_ = 3.76, ***P*** **<0.01**	*t*_64_ = 1.12, *P* = 0.27	*t*_64_ = 0.68, *P* = 0.50
2^nd^ generation**	Sex ratio	*z* = 0.81, *P* = 0.42	-	-
	No. of adults	*z* = 1.12, *P* = 0.27	*z*<0.01, *P* = 1.0	*z* = 0.98, *P* = 0.33
	Wet body weight	*t*_211_ = 6.35, ***P*** **<0.01**	*t*_211_ = 5.20, ***P*** **<0.01**	*t*_211_ = 1.78, *P* = 0.077
	Elytra length	*t*_69_ = 3.22, ***P*** **<0.01**	*t*_69_ = 0.94, *P* = 0.35	*t*_69_ = 0.87, *P* = 0.39

* The two groups of insects were reared on artificial diets with and without 0.003% rifampicin, and the antibiotic-treated insects were mostly *Nardonella*-free while the untreated insects were mostly *Nardonella*-infected (see table 2).

** The two groups of insects were reared on sweet potatoes without the antibiotic, and the offspring of the antibiotic-treated insects were all *Nardonella*-free while the offspring of the untreated insects all *Nardonella*-infected (see table 2).

*** In the 1^st^ generation, therefore, not exactly reflecting *Nardonella* infection but actually antibiotic treatment.

Statistically significant effects are highlighted by bold type.

### Ecological, evolutionary and applied perspectives

Molecular phylogenetic studies suggested that the *Nardonella*-weevil association has been maintained for over 125 million years [14], [37], which is suggestive of vital necessity of the *Nardonella* endosymbionts for the host weevils. In this study, however, the host weevils deprived of *Nardonella* infection could grow, become adult, survive and reproduce, although their fitness parameters were negatively affected (Figs. 2–4). From these results, at a glance, it seems that the *Nardonella* endosymbiont is certainly beneficial but not essential for the host *E. postfasciatus*. However, this view may need to be carefully re-considered in the context of natural ecology of the insect. The sweet potato, *Ipomaea batatas*, is a crop plant that has been bred by human to be highly nutritious and less toxic [50]. Outside the sweet potato fields, *E. postfasciatus* utilizes and reproduces on wild *Ipomaea* plants such as *I. pes-caprae* and *I. indica*, which are likely to represent original host plants of the insect [39]. It appears plausible that *Nardonella* infection is essential for the weevils on less nutritious and more toxic natural host plants, to which future ecological studies of the symbiotic association should be directed. Even when *Nardonella* elimination does not lead to fatal consequences, the smaller and slow-growing *Nardonella*-free insects might suffer drastic fitness reduction due to, for example, lower competitiveness for food resources, lower possibility of mating success, and higher susceptibility to natural enemies, which could result in substantial sterility of the aposymbiotic insects under natural conditions. Considering that endosymbiont-derived trace nutrients such as vitamins may be vertically transferred to eggs from maternal pool [52], it is conceivable that the negative fitness effects of *Nardonella* elimination would become more evident in subsequent generations.

*Nardonella* represents the most ancient, primary endosymbiont in the weevil superfamily Curculionoidea [14], [37], [38]. Meanwhile, phylogenetic studies strongly suggest that *Nardonella* has been replaced by different bacterial endosymbionts repeatedly in the evolutionary course of weevils. Well-documented examples are the grain weevils of the genus *Sitophilus* harboring *Sodalis*-allied gammaproteobacterial endosymbionts [48], and the acorn weevils of the genus *Curculio* associated with gammaproteobacterial endosymbionts *Curculioniphilus buchneri* [53]. Here we suggest the possibility that nutritional quality of the food source for the weevils might have affected the relationship to their endosymbionts and facilitated the endosymbiont replacements. Among the food stuffs utilized by weevils, rice and corn grains for *Sitophilus* species and chestnuts and acorns for *Curculio* species appear relatively nutritious in comparison with, for example, dead wood and other plant parts utilized by many dryophthorid and other groups of weevils [34], [35]. The better nutritional quality of their foods might have relaxed the natural selection acting on the endosymbiosis, attenuating the indispensability of the primary endosymbiont and facilitating the endosymbiont replacements. In this context, it is of ecological and evolutionary interest to examine whether endosymbiotic association with *Nardonella* is correlated with nutritionally deficient food sources utilized by the host weevils.

*E. postfasciatus* is a serious pest of sweet potatoes worldwide [39], and an eradication program on the basis of sterile insect technique has been promoted [40]. The mutualistic endosymbiont *Nardonella* could be a novel target for controlling the pest weevil. The artificial diet rearing system for *E. postfasciatus*, which was originally devised for mass-production of irradiated sterile weevils [41]–[43], provides a useful tool to investigate nutritional, physiological and molecular interactions underpinning the *Nardonella*-weevil endosymbiotic association.
